# Strides in Dental Education

**Published:** 1907-06

**Authors:** E. N. Kibler


### STRIDES IN DENTAL EDUCATION.

By Dr. E. N. Kibler, D. D. S.

S. C. State Dental Association, 1906.

To gain knowledge we must study the past and the present, by so doing we can aptly foretell the future. The men who make a success and are an honor to the profession, are those who have made diligent research and are constantly striving to place the practice of Dental Surgery to the fore; men who never tire in their efforts for improvement; men who take a pride in the advancement of the profession; men whose watchword is progress.

I may say that we have many men who call themselves dentists, that are mere mechanical fumblers, and are in the profession only for the monetary consideration. Such are a disgrace to the profession and a fraud on humanity. There is no profession that has made such wonderful strides of progress as ours in the last twenty-five years. We are now just blooming into the zenith of perfection.

We are standing side by side with our sister profession, medicine, and are reaping the honors that so justly belong to us, and are destined to be second to none. I believe that God in the creation of man placed within his mouth the thirty-two precious enameled jewels, as a gift for life. Have we any other organs composed of like structure. The tooth to an uneducated eye is a very simple object. But to the educated a momentous compact; what is more beautiful than the pearly luster of enamel, the protector of its sister dentine. It is needless for me to describe the three different structures and the pulp with its nerves, arteries, veins and lymphatics, to the educated dentist, he is fully acquainted.

Think of all the different compartments of vitality of the human body developed with the care, as that of a tooth and tell me it is only for a temporary period. I would sooner tell you a stone fort was built for a temporary purpose. Our mouth is the gateway of health, and our teeth the batteries that accomplish the first work of perfect digestion, that goes towards giving us a healthy body and apt mind.

To take care of these precious organs we need an educated Dental Surgeon. Which is a high and honorable calling, that no man with high attainments or aspirations need be ashamed of. I am glad to see the diversity of progress we are making. Take the branch and progress of Orthodontia and look at the beautifyings and marks of intelligence we have given our patients.

We are living in an age of Oslerism. When a man of broken down teeth, mal-occlusion, protrusion, etc., need not apply for a position of trust or honor. Because these deformities are marks of age and idiocy to the business public. But let this man go to an ethical orthordentist, who will regulate the deformities and replace the lost structures. He will then appear as a man of youth and intelligence.

I say we are meeting with honor and praise on all sides. The public is beginning to awake from a long sleep, to the object and physiological necessity of the teeth, their care and attention, so it behooves us as dentists to be well informed on all subjects pertaining to our profession. The day is fast coming when only the educated, ethical dentist will succeed; the quack will be unable to live upon the public, as the public is becoming more and more educated and will recognize him at a glance.

We can't be too loyal to our associations. We should be proud of them and honor them by our presence. They have been the nuclous of education, from which has radiated honor, distinction and beneficence. It is through our associations we have efficient and ethical members on the State Board, and the State Boards have stimulated the Dental Colleges to a higher ideal, and have run the purchasing diploma concerns out of business. We then should look forward to our annual meetings with pleasure, as a great dental summer school, where we exchange methods and ideas freely.

We as dentists should be a benefactor to the locality in which we reside. We should be careful in the advice we give young men who desire to study the profession.

So many seek the profession because they think it an easy road to wealth. We need no such, because usually they are the embodiment of quackery. But I think it our duty to the profession and public to advise young men with high moral character, education and mechanical ingenuity that would be an honor to the profession and a benefaction to the public. From such we can expect the full bloom of progressiveness of the dental profession, whereby it will attain the high recognition among the learned professions it justly deserves.

